# LncRNA-AK012226 Is Involved in Fat Accumulation in db/db Mice Fatty Liver and Non-alcoholic Fatty Liver Disease Cell Model

**DOI:** 10.3389/fphar.2018.00888

**Published:** 2018-08-08

**Authors:** Xingtian Chen, Yangzhi Xu, Dan Zhao, Ting Chen, Chengxin Gu, Ganxiang Yu, Ken Chen, Yun Zhong, Jie He, Shiming Liu, Yuqiang Nie, Hui Yang

**Affiliations:** ^1^Department of Gastroenterology, The Second Affiliated Hospital of Guangzhou Medical University, Guangzhou, China; ^2^Department of Cadre Health Care, Guangdong Pharmaceutical University, Guangzhou, China; ^3^Guangzhou Institute of Cardiovascular Disease, The Second Affiliated Hospital of Guangzhou Medical University, Guangzhou, China; ^4^Department of Gastroenterology, Guangzhou First People’s Hospital, Guangzhou Medical University, Guangzhou, China

**Keywords:** lncRNA, AK012226, non-alcoholic fatty liver, microarray, lipid accumulation

## Abstract

Instances of obesity and related metabolic abnormalities are increasing across the world. Non-alcoholic fatty liver disease (NAFLD) is a common disorder in obese people and is becoming the leading cause of hepatocellular carcinoma. Recently, long non-coding RNAs (lncRNAs) have been proven to play remarkable roles in numerous biological processes and human diseases, including NAFLD. However, the function of lncRNA in NAFLD pathogenesis remains largely unknown. The aim of this study was to explore the lncRNA expression profile in NAFLD mice and to identify novel lncRNAs involved in the pathogenesis of NAFLD. We performed microarray analysis to compare the expression profiles of lncRNAs and mRNAs in the liver of diabetic db/db mice with NAFLD and normal mice. A total of 3360 lncRNAs (2048 up-regulated and 1312 down-regulated) and 2685 mRNAs (1195 up-regulated and 1490 down-regulated) were found to be differentially expressed between the NAFLD and control groups. Real-time PCR validation of five differentially expressed lncRNAs in the liver samples was consistent with the microarray results. Besides, the up-regulated lncRNA, AK012226, was also significantly increased in an NCTC1469 NAFLD cellular model. Thus, the up-regulated lncRNA, AK012226, was chosen for subsequent studies. A co-expression network of AK012226-mRNAs was constructed and bioinformatic analysis of these co-expressed mRNAs indicated that they were enriched in the PPAR signaling pathway. Furthermore, Nile red staining and flow cytometry analysis revealed that knockdown of AK012226 by siRNA significantly reduced the lipid accumulation in the NCTC1469 cells treated with free fatty acids. In conclusion, the present study identifies the dysregulated lncRNAs and mRNAs involved in NAFLD, and in particular, a novel lncRNA, AK012226, was identified to be associated with lipid accumulation in NAFLD.

## Introduction

Instances of obesity and related metabolic syndrome are increasing around the world. According to the Global Health Observatory (GHO) data from World Health Organization (WHO), an estimated 600 million adults, accounting for approximately 13% of the world’s adult, were obese in 2014; this number has more than doubled since 1980 (WHO, 2014). Because of strong association with obesity, non-alcoholic fatty liver disease (NAFLD) has emerged as the most common chronic liver disease, worldwide. It is estimated that its prevalence is 20–30% in the general population and 70% in patients with type 2 diabetes and is strikingly increased to 90–95% in obese people (Non-alcoholic Fatty Liver Disease Study Group et al., 2015; Portillo-Sanchez et al., 2015; Younossi et al., 2016). Thus, NAFLD is regarded as the hepatic manifestation of metabolic syndrome. Moreover, NAFLD is a risk factor for hepatocellular carcinoma (HCC), accounting for up to 25% of the cases of HCC in the western countries, and has become one of the most important public health problems (Michelotti et al., 2013).

Non-alcoholic fatty liver disease includes a spectrum of diseases ranging from simple steatosis to steatohepatitis, advanced fibrosis, and cirrhosis (Anstee et al., 2011). However, the mechanism underlying the pathogenesis of NAFLD has not yet been fully elucidated and, thus, there are no effective medical interventions to completely cure NAFLD. Currently, the treatment for NAFLD remains limited, and mainly includes lifestyle alteration and body weight control; however, such strategies have poor compliance. A better understanding of the pathogenesis of NAFLD and devising of effective prevention and treatment modalities are, therefore, urgently required.

Long non-coding RNAs (lncRNAs) refer to RNA transcripts longer than 200 nucleotides that lack protein-coding capacity (Wilusz et al., 2009). Recently, emerging evidence has proved that lncRNAs play remarkable roles in numerous biological processes (Mercer et al., 2009; Wang and Chang, 2011; Wang et al., 2011; Rinn and Chang, 2012; Li et al., 2015), including cell differentiation, tumorigenesis, immune response, and hepatic lipid metabolism through transcriptional and epigenetic gene regulation. Currently, many lncRNAs are known to be involved in liver diseases. For example, H19, HULC, and HOTAIR are lncRNAs that are upregulated in the hepatocellular carcinomas and promote tumor growth through various mechanisms. Additionally, in a recent study, we identified a new lncRNA, uc002mbe.2, which plays a critical role in trichostatin A-induced apoptosis of HCC cells (Yang et al., 2013; Chen T. et al., 2017). Moreover, increasing evidence suggests that lncRNA may also play key roles in lipid metabolism. Li et al. (2015) identified a liver-enriched lncRNA, lncLSTR, which regulates apoC2 expression *via* FXR-mediated pathway. Depletion of lncLSTR increases apoC2 expression, initiating LPL activation and enhances serum triglycerides clearance (Li et al., 2015). However, the roles of lncRNAs in the pathogenesis of NAFLD remain largely unknown.

In this study, we performed a microarray analysis to compare the liver lncRNA and mRNA profiles in diabetic db/db mice and normal mice. Using several approaches for validation and mechanistic elucidation, we demonstrate that an lncRNA, AK012226, plays a crucial role in lipid accumulation and, thereby, in the pathogenesis of NAFLD.

## Materials and Methods

### Animal Studies

Three female C57BLKS/J db/db mice and three female C57BLKS/J mice aged 4 weeks, purchased from Changzhou Cavens Laboratory Animal, Co., China were used as the NAFLD and control groups, respectively. The mice were housed in a pathogen-free barrier facility with a 12 h light/dark cycle and received standard diet, and free access to water and food. At the age of 8 weeks, serum samples were collected prior to the sacrifice of mice and liver was harvested for further studies.

This study was approved by the Ethical Committee of Guangzhou Medical University, China. All the animal experiments complied with the standard ethical guidelines prescribed by the ethical committees mentioned above.

### Hematoxylin and Eosin Staining

Liver tissue samples were fixed in 4% paraformaldehyde in phosphate-buffered saline (PBS) for 2 h, stored overnight in 10% formalin, and were embedded in paraffin. Cross-sections (5 μm) of the tissue were cut and used for staining with hematoxylin and eosin. Images were taken using a light microscope.

### Measurement of Blood Glucose and Liver Triglyceride Levels

Sera were obtained by centrifugation of blood at 1500 ×*g* for 10 min after coagulation. The levels of blood glucose were measured with an automated analyzer for clinical chemistry (Arkray, Kyoto, Japan). The triglycerides in the liver were measured using the methods described in the kits from Nanjing Jiancheng Bioengineering Institute (Nanjing, China).

### RNA Preparation and Microarray Analysis

Total RNA was extracted from the liver of six mice (three NAFLD and control mice, each) using Trizol and treated with DNase I (Invitrogen, Carlsbad, CA, United States). The integrity of RNA was assessed by electrophoresis on denatured agarose gel. Mouse LncRNA Microarray V2.0 (Arraystar, Rockville, MD, United States) was used to study the profiles of mouse lncRNAs and protein-coding transcripts in the liver. Nearly 31,423 lncRNAs and 25,376 coding transcripts could be detected using second-generation LncRNA microarray. The LncRNAs are carefully collected from the most authoritative databases such as RefSeq, UCSC Knowngenes, Ensembl and many related literatures. Each transcript is represented by a specific exon or splice junction probe which can identify individual transcript accurately. Each sample was amplified and transcribed into fluorescent cRNA along the entire length of the transcripts without 3′-bias using a random priming method (Arraystar Flash RNA Labeling Kit, Arraystar). The labeled cRNAs were hybridized onto a Mouse lncRNA Array v2.0 (8 × 60K, Arraystar). After extensive washing, the arrays were scanned by an Agilent G2505C Scanner. The Agilent Feature Extraction software (version 11.0.1.1) was used to analyze the acquired array images. The GeneSpringGXv11.5.1 software package (Agilent Technologies) offered quantile normalization and background correction. The differentially expressed lncRNAs and mRNAs between the two samples were identified through fold change filtering. Pathway and gene ontology (GO) analyses were performed to determine the roles of the differentially expressed mRNAs in these biological pathways and the GO terms, respectively. Finally, hierarchical clustering was performed to obtain distinguishable LncRNA and mRNA expression patterns among the samples.

### RNA Extraction and Quantitative Real-Time PCR

Total RNA from liver tissue samples and cultured cells was isolated using Trizol and treated with DNase I (Invitrogen, Carlsbad, CA, United States). In brief, quantitative real-time PCR was carried out to study lncRNA expression using the Prime Script RT Reagent Kit (TaKaRa, Dalian, China) and SYBR Premix Ex Taq (TaKaRa, Dalian, China). Real-time PCR was performed on ABI Prism 7300 real-time PCR system (Applied Biosystems, Foster City, CA, United States). The quantification analysis for target gene expression was performed using the relative quantification comparative CT method. The primers used for the real-time PCR are listed in **Table 1**.

**Table 1:** Oligonucleotide sequences of the quantitative real-time RT-PCR primers.

Primer name	Sequence (5′→3′)
GAPDH	F: 5′ GTTGTCTCCTGCGACTTCA 3′ R: 5′ GCCCCTCCTGTTATTATGG 3′
AK012226	F: 5′ TCGTCTCCGAGCAGATTGTTG 3′ R: 5′ TCGTCTCCGAGCAGATTGTTG 3′
B430212C06Rik	F: 5′ TGGAGTCTTCCATTGGGAACTG 3′ R: 5′ TGGCTTGTCTCAGCAAAGATACTC 3′
Fabp3-ps1	F: 5′ TCCTCACTCATCGCACCATG 3′ R: 5′ AGCCCACACCGAGTGACTTC 3′
NR-040532	F: 5′ GAGAGACACTGGGACTGGCTTG 3′ R: 5′ TGTGAGGTGCTACAGAGTTGGTC 3′
AK052193	F: 5′ ACAAGTGAGTATCTGGGCTTTATCC 3′ R: 5′ GGTTTGTCCTCTGACCTCCACTC 3′
ENSMUST0000139794	F: 5′ AGAACGGATGACGCTGCCTC 3′ R: 5′ TGGCAGCCGTGTGGAACTAG 3′


### Nile Red Staining and Flow Cytometry Analysis

After the different treatments, cells were fixed with 4% paraformaldehyde at 26°C for 15 min, and incubated for 15 min with 1 μM Nile red dye working fluid (a hydrophobic dye that accumulates in lipid droplets). Thereafter, DAPI was added to stain the nucleus and the cells were examined by a confocal microscope (Carl Zeiss Company, Germany) or a digitized fluorescent microscope (BD Bio-sciences Pharmingen, San Diego, CA, United States). For quantitative analysis, cells were washed with PBS, centrifuged, and suspended in 500 μM Nile red dye stained for 15 min. After repeated centrifugation, the cells were incubated under 5% CO_2_ at 37°C for 15 min and were analyzed the fluorescence intensity of each group using flow cytometry (BD bio-sciences Pharmingen, San Diego, CA, United States).

### Cell Culture and Treatment

NCTC 1469 cells were bought from the Institute of Biochemistry and Cell Biology, Chinese Academy of Science (Shanghai, China) and cultured in Dulbecco’s modified Eagle’s medium (Mediatech, Herndon, VA, United States), supplemented with 10% (v/v) fetal bovine serum (Atlanta Biologicals, Lawrenceville, GA, United States) at 37°C under 5% CO_2_. The cells were treated with a mixture of 600 or 800 μM free fatty acids (FFAs; oleic acid:palmitic acid = 2:1) for 24 h to develop NAFLD cell model.

### siRNA Transfection

Scrambled siRNAs and predesigned siRNAs specific to different sites of lncRNA AK012226 were designed by the Robobio Company (Guangzhou, China). Cell transfection was conducted using Lipofectamine^TM^ RNAiMAX reagent (Invitrogen, Carlsbad, CA, United States) with lncRNA AK012226 siRNA. After 48 h, the cells were harvested to evaluate the efficiency of AK012226 lncRNA knockdown by quantitative real-time PCR.

### LncRNA–mRNA Co-expression Network

The lncRNA–mRNA co-expression network was constructed based on the correlation between the lncRNA-AK012226 and the differentially expressed mRNAs using Pearson’s correlation coefficient. The lncRNA–mRNA pairs with correlation coefficient significantly more than 0.85 were selected to construct the network (Pujana et al., 2007; Prieto et al., 2008).

### Statistical Analysis

Differentially expressed lncRNAs and mRNAs with statistical significance were identified through Volcano Plot filtering. The fold change > 2.0 and *p*-value < 0.05 was set for the threshold of up- and down-regulated genes. The data are presented as mean ± SD. Statistical analysis was performed using Student’s *t*-test for two-group comparison. Significance was defined as *p* < 0.05.

## Results

### Animal Model of Non-alcoholic Fatty Liver Disease

**FIGURE 1:**
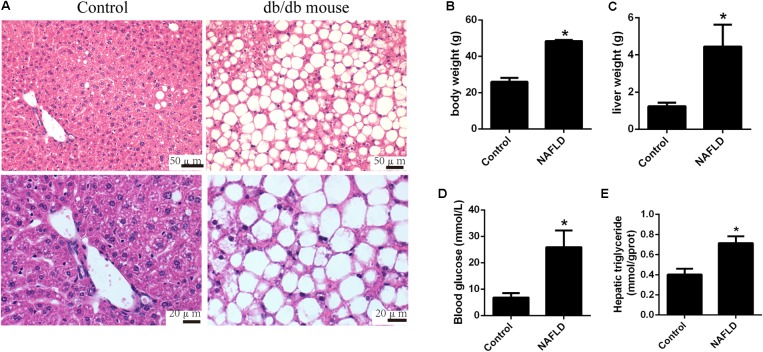
Pathological changes in db/db and control mice. **(A)** Representative images of the hematoxylin and eosin stained liver of db/db mice. **(B)** Body weight, **(C)** liver weight, **(D)** blood glucose level, and **(E)** hepatic triglyceride level of db/db and control mice. The values in bar graphs are means ± SD. ^∗^*p* < 0.05.

Due to the dysfunction of leptin receptor, the db/db mice develop hyperphagia-induced obesity, type 2 diabetes, and non-alcoholic fatty liver (NAFL) spontaneously. Obvious hepatic steatosis of db/db mice was observed by H&E staining (**Figure 1A**). Moreover, the body weight (48.40 ± 0.4899 vs. 25.93 ± 1.7913, *p* < 0.0001), the liver weight (4.443 ± 0.9686 vs. 1.237 ± 0.1621, *p* = 0.0099), the hepatic triglyceride level (0.7144 ± 0.0236 vs. 0.4016 ± 0.0206, *p* < 0.0001), and the blood glucose level (25.90 ± 5.1554 vs. 6.850 ± 1.3766, *p* = 0.0072) were significantly elevated in the db/db mice group compared to those in the control group (**Figures 1B–E**). Taken together, the db/db mice exhibited obesity, type 2 diabetes, and NAFL.

### Expression Profiles of lncRNAs and mRNAs in Mice With NAFL

**FIGURE 2:**
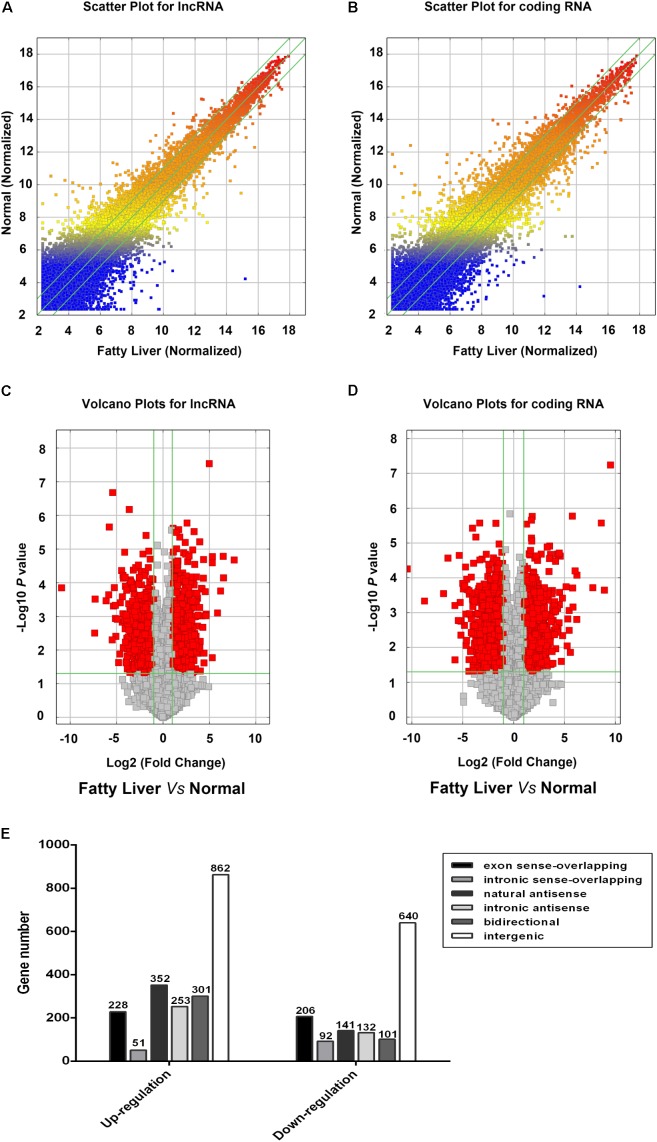
Profiles of lncRNAs and coding gene RNAs in non-alcoholic fatty liver disease (NAFLD) mice and control mice. Total RNA from three db/db mice and three control mice were isolated and gene expression was determined by Mouse LncRNA Microarray V2.0. The scatter plot was used for assessing the reproducibility of long non-coding RNA (lncRNA; **A**) and coding gene **(B)** expression. The values on x and y axis in the scatter plot are the averaged normalized signal values of groups of samples (log 2 scaled). Volcano plot analysis of the microarray chip data on differentially expressed lncRNAs **(C)** and coding gene **(D)** between the NAFLD and control mice. The vertical green lines correspond to a 2.0-fold up or down regulation whereas the horizontal green line represents a value of 0.05. The red dots to the left and right of the vertical green lines indicate more than 2.0-fold change and represent differential expression with statistical significance. Statistical significance was defined as fold change > 2.0 and *p*-value < 0.05 between the NAFLD and control groups. **(E)** Distribution of differentially expressed lncRNAs according to their classification.

**FIGURE 3:**
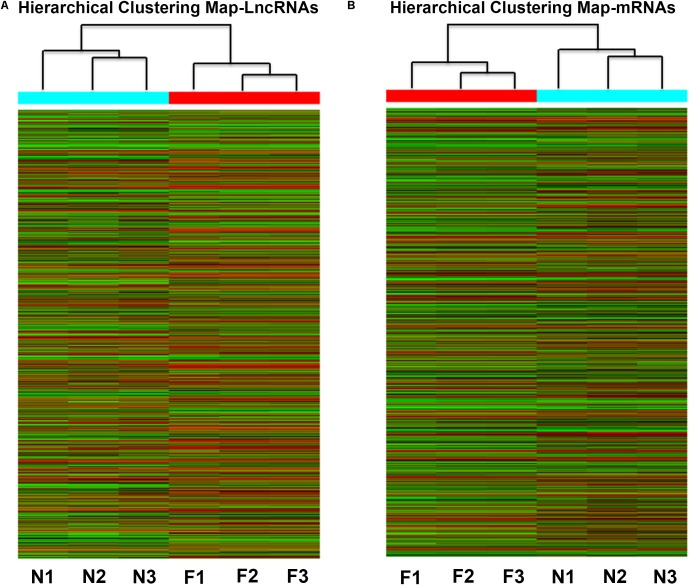
Heat map of the differentially expressed lncRNAs **(A)** and coding genes **(B)** in NAFLD mice and control mice. Each row represents the relative expression level of a single lncRNA or mRNA, and each column represents a single sample. High relative expression is indicated by red color and low relative expression is indicated by green color. F1-3: NAFLD group. N1-3: control group.

To determine the expression profiles of lncRNAs and mRNAs in mice with NAFL, the Mouse LncRNA Microarray V2.0 (Arraystar, Rockville, MD, United States) were used. The microarray data is available in Gene Expression Omnibus (GSE108228). The scatter plot and volcano plot analyses are shown in **Figure 2**. It was found that a total of 3360 lncRNAs (2048 upregulated and 1312 downregulated) and 2685 mRNAs (1195 upregulated and 1490 downregulated) were differentially expressed between the NAFLD and control groups. Hierarchical clustering was performed to analyze the gene expression patterns between the samples. A distinguishable pattern was observed between the NAFLD and control groups (**Figure 3**). Among these aberrant expressed lncRNAs, there were 228/206 exon sense-overlapping (up-/down-regulated lncRNAs), 51/92 intronic sense-overlapping, 352/141 natural antisense, 253/132 intronic antisense, 301/101 bidirectional, and 862/640 intergenic lncRNAs (**Figure 2E**).

### Validation of the Microarray Data Using Quantitative Real-Time PCR

**FIGURE 4:**
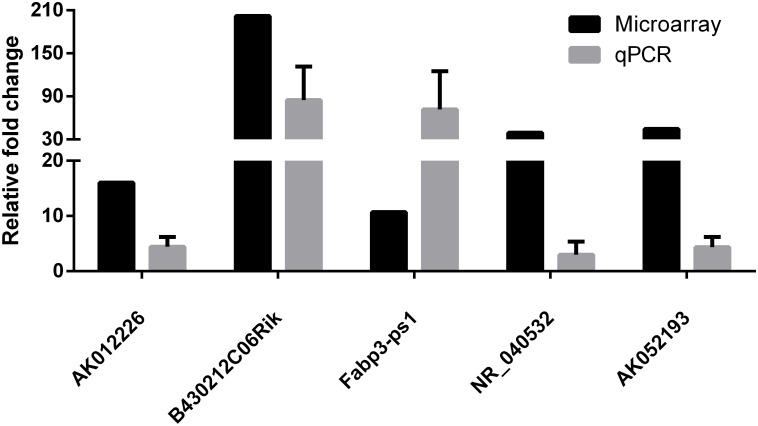
Comparison of microarray data and real-time quantitative polymerase chain reaction (qPCR) data for five selected differentially expressed lncRNAs in liver samples between NAFLD mice and control mice. The qPCR results are generally consistent with the microarray data. The results are the means of three independent experiments. The values in bar graphs are means ± SD.

To confirm the microarray results, five differentially expressed lncRNAs (AK012226, B430212C06Rik, Fabp3-ps1, NR-040532, and AK052193) were selected for qPCR validation in the liver samples of the NAFLD and control groups. As presented in **Figure 4**, the qPCR results were generally consistent with the microarray data, confirming the reliability of the microarray result (**Figure 4**).

**FIGURE 5:**
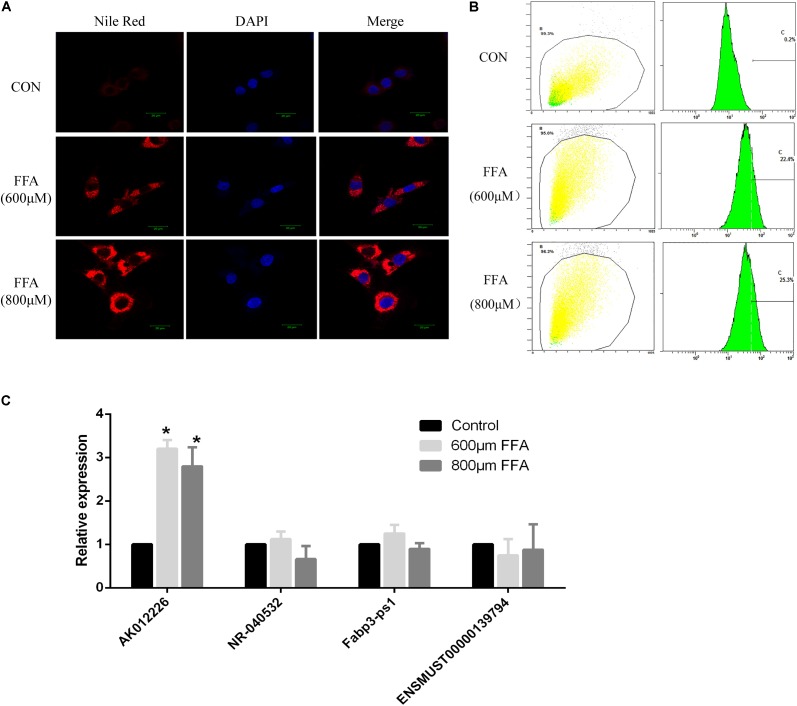
Expression of four differentially expressed lncRNAs in NCTC1469 cells treated with free fatty acids (FFAs). NCTC1469 cells were treated with FFAs at a concentration of 600 or 800 μM or dimethyl sulfoxide (DMSO) as control for 24 h. Obvious lipid accumulation was observed in FFA-treated cells compared to that in the control cells as detected by Nile red staining **(A)** and flow cytometry analysis **(B)**. **(C)** Quantitative real-time PCR of four up-regulated genes (AK012226, NR-040532, Fabp3-ps1, and ENSMUST00000139794) were performed to examine their expression levels. AK012226 expression was significantly increased in the NCTC1469 cells treated with FFAs. The results are the means of three independent experiments. The values in bar graphs are means ± SD. ^∗^*p* < 0.05.

In addition, we also examined the expression of differentially expressed lncRNAs in the mouse NAFLD cellular model. Murine NCTC 1469 hepatocytes were exposed to FFA at a concentration of 600 or 800 μM for 24 h to establish the *in vitro* NAFLD model. Obvious lipid accumulation, as detected by Nile red staining, was observed in cells treated with FFA compared to that in the control cells. This was further confirmed by flow cytometric analysis of the fluorescence intensity (**Figures 5A,B**). Subsequently, qPCR of four dysregulated lncRNAs (AK012226, NR-040532, Fabp3-ps1, and ENSMUST0000139794) was conducted in the mouse NAFLD cellular model. However, only the expression of the up-regulated lncRNA-AK012226 was consistent with the microarray data, whereas the others did not show differential expression (**Figure 5C**). Thus, the up-regulated lncRNA, AK012226, was chosen for subsequent experiments.

### Construction of AK012226-mRNA Co-expression Network and Bioinformatic Analysis

To ascertain the correlation between lncRNA AK012226 and the differentially expressed mRNAs, a co-expression network was built. A total of 218 mRNAs co-expressed with AK012226 with *p*-value < 0.05 and an absolute value of correlation coefficient > 0.85 was identified. The top 10 positively/negatively correlated mRNAs are listed in **Tables 2**, **3**.

**Table 2:** Top 10 positively correlated mRNAs.

Accession	Gene symbol	Definition	Correlation	*p*-Value
NM_001102578	Vmn2r75	Vomeronasal 2, receptor 75	0.9994003	0.002777778
NM_029116	Kbtbd11	Kelch repeat and BTB (POZ) domain containing 11	0.99860294	0.001388889
NM_008509	Lpl	Lipoprotein lipase	0.998057075	0.004166667
NM_007545	Hrk	BCL2 interacting protein (contains only BH3 domain)	0.997957619	0.001388889
NM_175340	Nhlrc1	NHL repeat containing 1	0.997585415	0.004166667
NM_009988	Cxadr	Coxsackie virus and adenovirus receptor	0.997267846	0.002777778
NM_212457	Bex4	Brain expressed X-linked 4	0.997135417	0.002777778
NM_001081651	Rab42	Member RAS oncogene family	0.99698408	0.004166667
NM_029011	Pyroxd2	Pyridine nucleotide-disulfide oxidoreductase domain 2	0.996300767	0.001388889
NM_001033148	1700029J07Rik	RIKEN cDNA 1700029J07 gene	0.995910932	0.004166667


**Table 3:** Top 10 negatively correlated mRNAs.

Accession	Gene symbol	Definition	Correlation	*p*-Value
NM_009502	Vcl	Vinculin	-0.955008039	0.016666667
NM_133203	Klra17	Killer cell lectin-like receptor, subfamily A, member 17	-0.94838445	0.033333333
NM_133882	C8b	Complement component 8, beta polypeptide	-0.944792307	0.038888889
NM_172306	Cyp4a12b	Cytochrome P450, family 4, subfamily a, polypeptide 12B	-0.9397872	0.030555556
NM_007912	Egfr	Epidermal growth factor receptor	-0.938161248	0.047222222
NM_177406	Cyp4a12a	Cytochrome P450, family 4, subfamily a, polypeptide 12a	-0.934294503	0.047222222
NM_153601	Lgsn	Lens protein with glutamine synthetase domain	-0.929686267	0.041666667
NM_001159415	Ces3b	Carboxylesterase 3B	-0.926505763	0.025
NM_177620	Rin3	Ras and Rab interactor 3	-0.920528278	0.048611111
NM_001081174	Rsg1	REM2 and RAB-like small GTPase 1	-0.919993451	0.029166667


**FIGURE 6:**
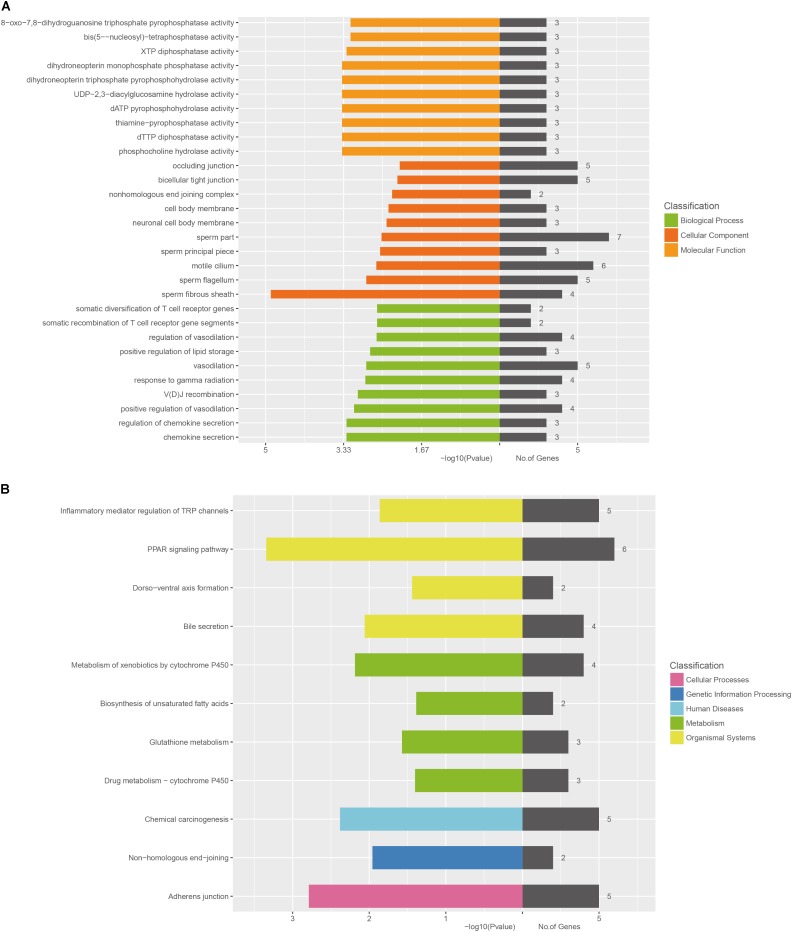
Bioinformatic analysis of mRNAs co-expressed with lncRNA-AK012226. **(A)** Gene ontology (GO) enrichment analysis of the co-expressed mRNAs. **(B)** KEGG pathway analysis of the co-expressed mRNAs. The horizontal axis represents -lg (*p*-value) of the GO terms and the pathway terms.

The GO categories for each gene were derived from the GO website^1^. The categories comprised of three structured networks: biological processes, cellular components, and molecular function. Through the analysis of the GO terms, it was found that the co-expressed mRNAs were principally enriched in the following components: (1) chemokine secretion, (2) regulation of chemokine secretion, (3) positive regulation of vasodilation, (4) V(D)J recombination, and (5) response to gamma radiation in biological process. The top10 GO terms in biological process, cellular components, and molecular functions are shown in **Figure 6**.

The KEGG pathway analysis was also performed using the KEGG database^2^. The co-expressed mRNAs were associated with “PPAR signaling pathway,” “adherens junction,” “Chemical carcinogenesis,” “Metabolism of xenobiotics by cytochrome P450,” and “Bile secretion.”

### LncRNA AK012226 Participates in the Development of NAFL in the Cellular Model

**FIGURE 7:**
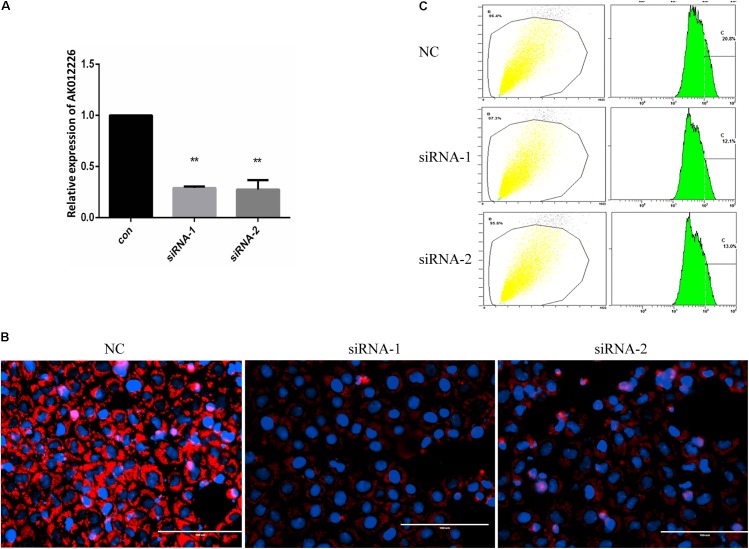
Knockdown of AK012226 expression significantly decreased the lipid accumulation in NCTC1469 cells treated with FFAs. **(A)** AK012226 expression in NCTC1469 cells transfected with two siRNAs targeting different sites of AK012226, respectively, followed by 24-h FFA treatment. **(B)** AK012226 siRNA-transfected NCTC1469 cells were exposed to FFAs for 24 h and were subsequently examined by Nile red staining. **(C)** Flow cytometric analysis of fluorescence in transfected NCTC1469 cells treated for 24 h with FFAs and subsequently stained with Nile red. ^∗∗^*p* < 0.01.

To further explore the role of AK012226 in the development of NAFL, NCTC 1469 cells were transfected with two AK012226-siRNAs targeting different sites and were subsequently treated with 800 μM FFA. The qPCR results showed that the AK012226 expression was knocked down by about 70% in the transfected cells (**Figure 7A**). Next, we examined the cellular lipid droplets in the transfected NCTC 1469 cells exposed to 800 μM FFA. A striking decrease of lipid accumulation was observed in the transfected cells compared to that in the control cells by Nile red staining; this was further confirmed by flow cytometric analysis (**Figures 7B,C**). Taken together, the knockdown of AK012226 expression decreased the lipid accumulation in the FFA-treated NCTC cells, suggesting that AK012226 could play a functional role in the pathogenesis of NAFLD.

## Discussion

Long non-coding RNAs participate in various biological processes, and the aberrant lncRNA expression can cause diverse diseases, especially cancers. Increasing evidence suggests that lncRNAs can act as oncogenes or tumor suppressor RNAs (Gibb et al., 2011; Shi et al., 2013). However, as of date, little is known about the roles of lncRNA in the pathogenesis of NAFLD. In the present study, we performed genome-wide lncRNA and mRNA microarray analyses of liver samples from diabetic NAFL mouse to identify novel lncRNAs involved in the pathogenesis of NAFLD, aimed at obtaining new insights for the development of new therapeutic approaches. Notably, we discovered a novel lncRNA, AK012226, which was up-regulated in the liver of the NAFL mouse and was observed to play a role in the lipid accumulation *in vitro*.

Thus far, only a few studies have explored the role of lncRNAs in NAFLD. Sun et al. (2015) performed microarray experiments to analyze the profile of mRNAs and lncRNAs in patients with NAFLD, and 1735 lncRNAs and 1485 mRNAs were found to be differentially expressed in the NAFLD samples. A research from China (Chen Y. et al., 2017) involved microarrays in the high fat diet-induced NAFLD mice model and revealed several lncRNAs and mRNAs associated with lipogenesis through different pathways. Additionally, Chen et al. (2016) reported that lncRNA SRA could decrease lipolysis by repressing the expression of ATGL, leading to hepatic. However, the expression profiles of lncRNAs and mRNAs in our study were a bit different from those observed in the above-mentioned studies, which could be attributed to the different NAFLD model employed by us. Unlike the dietary mouse model of NAFLD used in several studies to perform genome wide analysis (Chen et al., 2016; Chen Y. et al., 2017), we employed the diabetic db/db mouse to establish the NAFLD animal model. Insulin resistance, frequently found in obesity and type 2 diabetes, is considered to be the most important factor in the pathogenesis of hepatic steatosis. Therefore, the animal model is a better for metabolic abnormalities, such as obesity, type 2 diabetes, and NAFLD (Sanches et al., 2015). The db/db mouse, defective in the leptin receptor gene, is a classic diabetic rodent model, manifesting hyperphagia, obesity, hyperlipidemia, insulin resistance, and hepatic steatosis (Tesch and Lim, 2011; Kitada et al., 2016). In the present study, our results indicated that the db/db mouse develop NAFL, which is consistent with the results obtained in other studies (Kim et al., 2015; Su et al., 2016). Thus, the db/db mouse is a proper animal model that mimics the metabolic syndrome-associated NAFLD in humans and is a suitable model to examine the role of lncRNAs in the pathogenesis of NAFLD.

Notably, we identified a novel lncRNA, AK012226, which plays a functional role in lipid accumulation in the mouse NAFLD cellular model. AK012226, located on the sense strand on chromosome 14 of mouse, is an exon sense-overlapping lncRNA. Exon-sense overlapping lncRNA is an lncRNA category that can be considered as transcript variants of protein-coding mRNAs, as they overlap with a known annotated gene on the same genomic strand. The gene predicted to be associated with it is 1700112E06Rik. However, this gene is considered to be involved in melanocyte differentiation. Moreover, our results indicated that the knockdown of AK012226 expression significantly reduced the *in vitro* lipid accumulation. Hepatic steatosis has long been considered as a relatively benign state in NAFLD. Recent studies have proven that fatty liver is more vulnerable to injury from various causes (Adams et al., 2005). Hepatic lipid accumulation is also associated with the increase in endoplasmic reticulum stress markers (Neuschwander-Tetri, 2010). The accumulation of lipid can further aggravate the existing hepatic insulin resistance by generation of lipid-derived second messengers, such as diacylglycerol (DAG) and ceramides (Jornayvaz and Shulman, 2012). Thus, it is considered as the “first hit” in the pathogenesis of NAFLD (Day and James, 1998). Therefore, lipid overaccumulation within hepatocytes is crucial in NAFLD (Donnelly et al., 2005; Reccia et al., 2017).

Recently, several lncRNAs involved in lipid metabolism have been identified. SPRY4-IT1, an lncRNA overexpressed in melanoma cells, negatively regulates triacylglycerol levels in melanoma cells and knockdown of SPRY4-IT1 can induce apoptosis *via* lipin 2-mediated alterations in lipid metabolism leading to cellular lipotoxicity (Mazar et al., 2014). In addition, a recent study identified a novel human lncRNA, lncHR1, which can decrease lipid accumulation both *in vitro* and *in vivo* by repressing the expression of SREBP-1c gene (Li et al., 2017). According to the results of co-expression network of AK012226–mRNAs, and the subsequent KEGG pathway and GO analyses, we found some pathways and GO terms to be likely linked to the pathogenesis of NAFLD, such as PPAR signaling pathway, biosynthesis of unsaturated fatty acids, bile secretion, and chemokine secretion. However, the underlying molecular mechanism of AK012226 in regulating lipid accumulation and in the pathogenesis of NAFLD has not yet been elucidated. Further investigation is needed to examine the role of AK012226 in the development of NAFLD.

## Conclusion

We identified differentially expressed lncRNAs and mRNAs in diabetic NAFLD mice. Particularly, we identified a novel lncRNA, AK012226, this lncRNA was found to be closely related to lipid accumulation in the NCTC1469 NAFLD cell model.

## Author Contributions

XC, YX, and DZ generated data, data analysis, interpretation, and manuscript preparation. TC generated data, perform animal experiment, and data analysis. CG, GY, and KC data analysis, interpretation, and manuscript preparation. YZ analyzed data and generated figures and tables. JH data analysis, interpretation, and manuscript preparation. SL data analysis, interpretation, and manuscript preparation. YN data analysis, interpretation, and manuscript preparation. HY generated idea, study design, data analysis, interpretation, and manuscript writing.

## Conflict of Interest Statement

The authors declare that the research was conducted in the absence of any commercial or financial relationships that could be construed as a potential conflict of interest.

## Footnotes

1 www.geneontology.org

2 http://www.genome.jp/kegg/
